# Altered expression of 3´paralogus *HOX A-D* clusters in endometriosis disease: A case-control study

**Published:** 2018-09

**Authors:** Masoumeh Golestan Jahromi, Reza Aflatoonian, Parvaneh Afsharian, Samaneh Aghajanpour, Maryam Shahhoseini, Abbas Aflatoonian

**Affiliations:** 1 *Research and Clinical Center for Infertility, Yazd Reproductive Sciences Institute, Shahid Sadoughi University of Medical Sciences, Yazd, Iran.*; 2 *Department of Genetics, Reproductive Biomedicine Research Center, Royan Institute for Reproductive Biomedicine, ACECR, Tehran, Iran.*; 3 *Department of Endocrinology and Female Infertility, Reproductive Biomedicine Research Center, Royan Institute for Reproductive Biomedicine, ACECR, Tehran, Iran.*; * Maryam Shahhoseini and Abbas Aflatoonian are an equal Corresponding Authors.

**Keywords:** Endometriosis, HOX genes, Eutopic endometrium, Ectopic endometrium

## Abstract

**Background:**

Endometriosis is a prevalent gynecological disease, with limited known etiology and more researches are required to identify its etiology. In this manner, there is no evidence for expression and function of 3´*HOX* genes in 4 clusters in the limb and pelvic organs such as the uterus and its disorders (Genes in the *HOXA*-*D* clusters are subdivided into 13 paralogous groups).

**Objective:**

This study designed to investigate the expression profile of 5 paralogous (1-5) in four clusters of *HOX* genes (A, B, C, and D) in ectopic and eutopic tissues of women with endometriosis compared to the normal endometrium.

**Materials and Methods:**

Samples were obtained from thirty patients (15 with and 15 without endometriosis) of reproductive age with normal menstrual cycles. The same patient provided both eutopic and ectopic tissues and control women were laparoscopically checked for the absence of endometriosis. The expression profile of these *HOX* genes was investigated by quantitative real-time polymerase chain reaction technique.

**Results:**

We observed significant up-regulation of some members of *HOXC* and *D* clusters (*HOXD1*, *HOXD3*, *HOXC4* and *HOXC5*) in ectopic and eutopic tissues vs. control. Also, our data showed significant down-regulation of all of *HOXA* and *HOXB* paralogous except *HOXA1* in ectopic tissues versus control.

**Conclusion:**

Our data showed specific cluster dependent modulation of the *HOX* genes expression in endometriosis (over-expression of some *HOX* genes in cluster *C* and D and down-regulation of *HOX* genes in cluster *A* and *B*) in ectopic and eutopic tissues compare to control group. Therefore, it is possible that change of expression level of these genes in endometrium plays a role in the pathogenesis of endometriosis.

## Introduction

Endometriosis is a gynecological disease, demonstrate by the presence of endometrial glands and stroma located in ectopic sites such as pelvic, peritoneum, ovaries, and rectovaginal septum. The most recognizable signs of the disease is pain such as chronic dysmenorrhea, intermenstrual abdominal and pelvic pain and back pain (1). The prevalence of endometriosis in the population is 6-10%; but in women with pain, infertility or both, increases to 35-60% (2). Endometriosis is considered as a complicated disorder and despite its high frequency; the etiology of the disease is not well understood. Moreover, the diagnostic and therapeutic approaches are not definitely known and more investigations are needed to identify its etiology (3).

Recent studies have suggested that abnormalities in the regulation of specific genes are involved in the incidence of endometriosis (4-7). In women with particular genetic backgrounds, specific networks of genes are involved in endometrial tissue formation (8). During mammalian evolvement, specification of various parts of body along the axis, from the branchial area through to the tail, is controlled by *HOX* genes (9). 

In vertebrates, the 39 *HOX* genes are classified into four clusters according to their location on the chromosomes: *HOXA, HOXB, HOXC* and *HOXD* (10). Scientists subdivided *HOX *genes within the *HOXA*-*D* clusters into 13 paralogous groups. Genes in each paralogous group have functional similarity with corresponding paralogous genes in other clusters (11). *Duboule* and colleagues determined that the expression pattern of *HOX* genes are sequentially from 3′ to 5′ along the anterior-posterior (AP) axis, during embryonic development, and the 3′ genes are expressed prior to the 5′ genes (12).

Each adult organ displays a specific grouping of *HOX* genes expression representing the molecular portrait (projection) of the organ (13). Furthermore, specific sets of homeobox genes regulate reproductive function in the adult (14), for example they have an important function in the regulation of cyclic endometrial regeneration (15). Furthermore*, HOXA10* and *HOXA11* regulate endometrial receptivity and also *HOXC* and *HOXD* genes have a role in the early development of endometrium and endometrial proliferation. Therefore, the network of *HOX* genes may be involved in multiple aspects of endometrial development and function, such as proliferation and differentiation (16). Due to this background, it is supposed that *HOX* genes have a functional role in endometriosis development. 

The aim of this study was identifying the expression pattern of 1-5 paralogous *HOX* genes in endometriosis (both eutopic and ectopic tissues) and comparing it to the endometrium of healthy women.

## Materials and methods

**Participants and tissue collection**

This case-control study was performed at the Research and Clinical Center for Infertility, Yazd, Iran and Royan Institute, Tehran, Iran. Fifteen women scheduled for surgery of endometriosis participated in this study. To minimize the genetic heterogeneity, one sample of endometriomas and one sample of eutopic endometrium have been collected from each participant in the proliferation phase. Also, fifteen healthy women were enrolled in this study as the control group. These women who confirmed to have no other causes of infertility were laparoscopically checked for the absence of endometriosis. 

Women participated in this study had normal menstruation cycle, not receiving hormone therapy from 3 months before the surgery and were younger than 37 yr. All patients were in the follicular phase of the menstrual cycle at the time of the surgical procedure. Samples of endometriosis tissue were obtained from ectopic sites in the abdomen; all samples used in this study were endometriomas. In each patient, the cyst wall of the endometrioma was removed during laparoscopy. Eutopic endometrial tissues were obtained from the uterus of the same patients at the time of the laparoscopy by performing hysteroscopy, followed by dilatation and curettage. Samples of the control group were attained during the hysteroscopy by curettage.

All of the samples were immediately placed in RNA protection reagent, RNAlater (Ambion, Austin, TX), frozen in liquid nitrogen and stored at -80^o^C. 

**RNA extraction and cDNA synthesis**

QIAGEN RNAeasy kit (QIAGEN, Venlo, the Netherlands) was used to extract mRNA according to the manufacturer’s instructions. RNA quality and concentration were measured using Nanodrop 2000 (Thermo, USA). cDNA synthesis was performed with the Super Script double-stranded cDNA synthesis kit (QIAGEN, Venlo, The Netherlands). Two thousand nanograms of total RNA were used for cDNA synthesis according to the manufacturer’s instruction of cDNA synthesis kit (Qiagen, Cat.No:207045). RT-PCR was performed with twenty-five nanogram of cDNA and human-specific primers. An oligonucleotide primer for specified amplification of each candidate gene was designed and tested. The sequences and annealing temperatures for each primer are listed in table I.

**Real-time polymerase chain reaction**

Quantitative polymerase chain reaction (q-PCR) was performed on the prepared cDNA samples with the use of primers designed for *HOXA1*, *HOXA2*, *HOXA3*, *HOXA4*, *HOXA5*, *HOXB1*, *HOXB2*, *HOXB3*, *HOXB4*, *HOXB5*, *HOXC4*, *HOXC5*, *HOXD1*, *HOXD3*, and *HOXD4*. A melting curve was generated after each run to verify the specificity of the primers, shown by the presence of a single band and no primer-dimer artifacts. Each reaction of the PCR plate contained 10 µL SYBR green (PCR Master Mix, 5 µL; Applied Biosystems), 6 µL molecular water, 1 µL of each primer (20 pmol), and 2 µL cDNA (final concentration equal to 25 nanograms per µL). Real-time PCR was performed under standard conditions, and all experiments were run in triplicate. The quantitative PCR data were analyzed with the use of the comparative cycle time (CT) method (17). The difference in cycle times, ΔCT, was determined as the difference between the tested gene and the housekeeping gene (β-actin). Then the ΔΔCT was obtained by finding the difference between patients and control groups. 

Reactions with a threshold cycle >35 were considered to be undetectable. Fold-change ratios between groups were derived and the 2-fold difference was applied to select up-regulated (fold ≥2) and down-regulated (fold ≤2) genes. The relative quantity of gene of interest was normalized to the relative quantity of β-actin, as reference gene and was reported as fold change of gene expression.

**Ethical consideration**

The study protocol was approved in both Yazd Research and Clinical Center for Infertility and ROYAN Institute ethics committees (EC/91/1131). Informed consent was obtained from all participants for the use of their tissue samples.

**Statistical analysis**

The values were expressed as mean±SEM. The data were analyzed by one-way ANOVA using SPSS (Statistical Package for the Social Sciences, version 21.0, SPSS Inc, Chicago, Illinois, USA) software, followed by Tukey’s test analysis to compare various groups with each other. The statistical significant difference was defined as p <0.05.

## Results

Expression of 3´paralogus genes in *HOXA* (1, 2, 3, 4, and 5), *HOXB* (1, 2, 3, 4, and 5), *HOXC* (4, and 5), and *HOXD* (1, 3, and 4) clusters were quantitatively evaluated in normal endometrium of healthy women, compare to eutopic and ectopic tissue of endometriosis patients.

In the way, *HOXA3*, *HOXA4*, and *HOXA5* showed significant down-regulation in ectopic group compare to the control group (p=0.0002, 0.0049, and 0.0032 respectively), also there was significant down-regulation in eutopic group compare to the control group (p=0.009, 0.0052, and 0.0033), *HOXA2* was significantly down-regulated in ectopic group compare to the eutopic and control group (p=0.0045, and 0.0050 respectively), however, there was no significant difference in mRNA expression of *HOXA1* between all groups.

**Figure 1:**
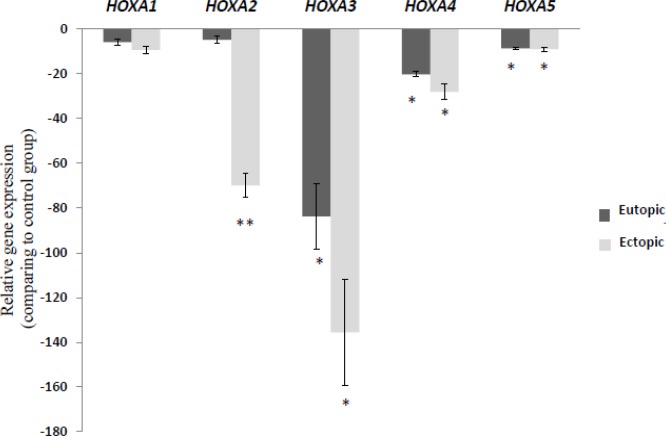
Relative gene expression of *HOXA* cluster genes in ectopic and eutopic tissues of endometriosis patients (n=15) compare to healthy women (n=15). The relative quantity of gene of interest was normalized to the relative quantity of *β-actin*, as reference gene and was reported as fold change of gene expression. Values expressed as mean ± SEM. Data were analyzed by ANOVA followed by Tukey’s multiple comparison test. (*p<0.05 compared to control group, ** p<0.05 between eutopic and ectopic groups).

**Figure 2:**
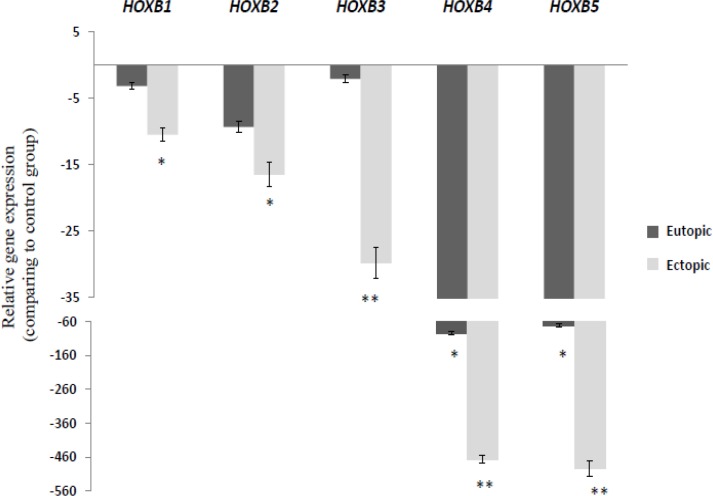
Relative gene expression of *HOXB* cluster genes in ectopic and eutopic tissues of endometriosis patients (n=15) compare to healthy women (n=15). The relative quantity of gene of interest was normalized to the relative quantity of *β-actin*, as reference gene and was reported as fold change of gene expression. Values expressed as mean±SEM. Data were analyzed by ANOVA followed by Tukey’s multiple comparison tests. (*p<0.05 compared to control group, ** p<0.05 between eutopic and ectopic groups).

*HOXA* cluster genes showed an overall down-regulation pattern in eutopic and ectopic groups compare to the control group (Figure 1); similarly, genes in the *HOXB* cluster showed significant down-regulation in eutopic and ectopic groups compare to the control group (Figure 2). 

*HOXB1*, *2*, *3*, *4*, and *5* was significantly down-regulation in ectopic group compare to the control group (p=0.003, 0.0021, 0.004, 0.0006, and 0.0002 respectively). Two *HOX* genes in B cluster (*HOXB4* and *HOXB5*) showed significantly down-regulation in eutopic groups compare to the control group (p=0.0007 and p=0.0002 respectively). Also, our data showed a significant decrease in the expression level of *HOXB3*, *HOXB4*, and *HOXB5* in ectopic tissues compare to the eutopic endometrium (p=0.0045, 0.0083, and 0.0076 respectively). Interestingly, the expression level of all *HOX* paralogous in this cluster was significantly lower in ectopic tissue compare to the eutopic endometrium. 

**Figure 3:**
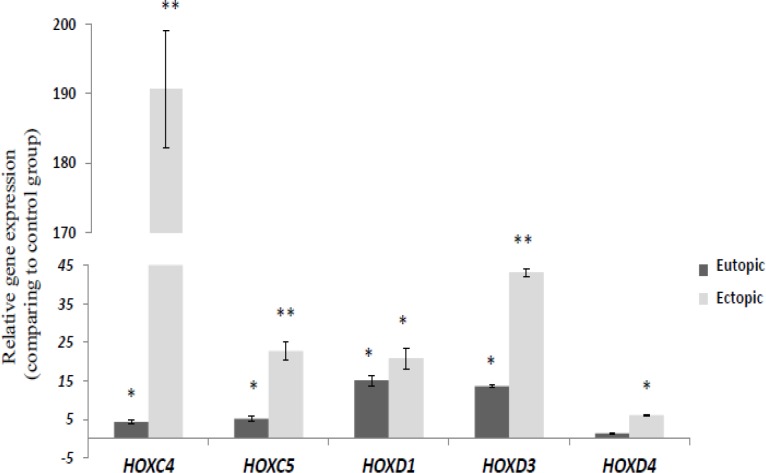
Relative gene expression of *HOXC *and* D* cluster genes in ectopic and eutopic tissues of endometriosis patient (n=15) compare to healthy women (n=15). The relative quantity of gene of interest was normalized to the relative quantity of β-actin, as reference gene and was reported as fold change of gene expression. Values expressed as mean±SEM. Data were analyzed by ANOVA followed by Tukey’s multiple comparison tests. (*p<0.05 compared to control group, ** p<0.05 between eutopic and ectopic groups).

In *HOXC* cluster, 3´ paralogous genes showed overall significant up-regulation in eutopic and ectopic group compare to the control group. There was a significant increase in mRNA expression level of *HOXC4* and *HOXC5* in ectopic tissues compare to the eutopic endometrium (p=0.0005 and p=0.006 respectively) (Figure 3). *HOXC4* and *HOXC5* were significantly over-expressed in ectopic compared to control group (p=0.0002 and 0.0005 respectively), also, there was a significant increase in mRNA expression level of *HOXC4* and *HOXC5* in eutopic group compare to the control group (p=0.019 and 0.0033 respectively). *HOXD1* and *HOXD3* genes also showed significant up-regulation in eutopic and ectopic group compare to the control group (p<0.05, Figure 3). *HOXD4* was significantly over-expressed in ectopic compare to control group, but there were no significant changes in its mRNA level in the eutopic tissues compared to the control group.

**Table I:** Sequence of the primers used for qPCR experiments

**Gene**	**Forward primer (F) Reverse primer (R)**	**Product size (bp)**	**Access number**
*β -actin*	F: 5' CAAGATCATTGCTCCTCCTG 3' R: 5' ATCCACATCTGCTGGAAGG 3'	151	NM_001101.4
*HOXA1*	F: 5' ACCCACCAAGAAGCCT 3' R: 5' TACTCTCCAACTTTCCCTG 3'	113	NM_153620.2
*HOXA2*	F: 5' AGGAGGACGAGGAAGAGA 3' R: 5' ACTGGGAAACTTTGGGAG 3'	151	NM_006735.3
*HOXA3*	F: 5' TTCCTGCCCTTTCCTTC 3' R: 5' CCATTCCAGCAACCAAGAT 3'	125	NM_153631.2
*HOXA4*	F: 5' AGGATGAAGTGGAAGAAAGA 3' R: 5' GGGATGGAGGTGTGGG 3'	121	NM_002141.4
*HOXA5*	F: 5' GAGCCACAAATCAAGCAC 3' R: 5' CGCCGAGTCCCTGAAT 3'	148	NM_019102.3
*HOXB1*	F: 5' AAACCCACCCAAGACAG 3' R: 5' GCAATCTCCACCCTCC 3'	150	NM_002144.3
*HOXB2*	F: 5' GCCACGTCTCCTTCTC 3' R: 5' CTTCTCCAGTTCCAGCAG 3'	150	NM_002145.3
*HOXB3*	F: 5' TTCCCTTCCAACTTTCCCA 3' R: 5' CACCATCCCTGAGCCC 3'	151	NM_001330323.1
*HOXB4*	F: 5' AGACAGAAAGAGAAATAGGAGG 3' R: 5' CGGCAGAGGAAACAAGAC 3'	120	NM_024015.4
*HOXB5*	F: 5' TATACCCGCTACCAGACC 3' R: 5' GTTGTCCTTCTTCCACTTCAT 3'	159	NM_002147.3
*HOXC4*	F: 5' TCCTCTCCCTCCCACT 3' R: 5' CCAGACCATCACACCTTG 3'	159	NM_014620.5
*HOXC5*	F: 5' TCAAAGAGTCACAAATCACC 3' R: 5' TCCATAGTTCCCACAAGTT 3'	148	NM_018953.3
*HOXD1*	F: 5' GCTTTCCAGACCGCATC 3' R: 5' GGCATTCCTCTTCACTTTCA 3'	114	NM_024501.2
*HOXD3*	F: 5' AAAGAAACCTGAAGAGCCT 3' R: 5' CACTGCCACCTCCAATG 3'	141	NM_006898.4
*HOXD4*	F: 5' CGGAGGATGAAGTGGAAA 3' R:5'CGTGTGGTGGTCTTTGG3'	138	NM_014621.2

## Discussion

Endometriosis still remains a significantly under-diagnosed and under-treated disease (18). There are numerous individual and public health concerns about the endometriosis and its treatment. Therefore, it is very important to understand its pathogenesis to help the prevention of the disease and also development of new and effective treatment strategies (19). The current study was undertaken to identify differentially expressed 3´ *HOX* genes paralogous in all four clusters in endometriosis compare to normal endometrium. An important finding of this study is a systematic alteration pattern of *HOX* genes clusters in endometriosis based on their cluster (down-regulation of *HOXA*, *HOXB* and up-regulation of *HOXC* and *HOXD*) in ectopic and eutopic tissue. Interestingly, during the current study we detected the meaningful difference levels in the expression of 3´ *HOX* gene members in the endometrium of the study groups. As there were more than 150-fold change in *HOXC* cluster besides more than 400-fold change in *HOXB* cluster. While we expected a very low expression level of these paralogous *HOX* genes in uterus and endometriosis implants due to their loci on chromosome. In addition, these genes specify and limited to the head and neck (20, 21) and naturally have not an important role in uterus development or etiology of related disease. *HOX* genes within the different clusters are classified as belonging to the one of 13 paralogous groups (*Hox1*-*Hox13*), based on their sequence similarities and relative positions in the loci, and a single cluster contains only a subset of the 13 groups. The spatial and temporal expression of *HOX* genes along the anterior-posterior axis during embryonic development is highly related to their physical organization (22, 23). Numerous studies investigated the role of *HOX* genes in endometriosis (15, 24, 25). Most of them explored the paralogous of 9 -13 in each cluster (26, 27). According to the cohort of studies, there is no evidence for definite expression and function of all 3´*HOX* genes in 4 clusters in the limb and pelvic organs such as the uterus. The majority of these *HOX* genes paralogous are expressed in the central nervous system (CNS), where they have critical functions in neuronal specification and target connectivity (20, 21). However during the current study, we detected the expression of 3´ *HOX* genes in the endometrium, with high levels of changes in expression of these members between study groups. These data hypothesized the functional role of 3´ paralogous *HOX* genes in uterus organogenesis and endometriosis pathology. Finally, according to our data, the altered expression of specific *HOX* genes appeared in ectopic and eutopic endometrial tissues compared to the normal endometrium. These mutual features between eutopic endometrium and ectopic tissue which are not detected in the endometrium of women without endometriosis may approve the hypothesis that the main defect inducing endometriosis such as alteration of gene expression could be occur in the eutopic endometrium or in the uterus.

## Conclusion

In summary, despite recent progress in the investigation of the different features of endometriosis, it still remains a debilitating disorder that affecting a large cohort of women. Our study showed that 1-5 paralogous *HOX* genes have different expression pattern in endometriotic tissue compare to eutopic and control tissues. Therefore our result revealed some unknown aspect of genes expression aberration in endometriosis. It is to be expected that new therapies will be established based on the molecular targets summarized above.

## Note

***This article has been extracted from Ph.D. thesis. (Masoumeh Golestan Jahromi)***
